# Non obstructive azoospermia as the only indicator of focal segmental glomerulosclerosis and chronic kidney failure: A case report

**DOI:** 10.1016/j.eucr.2025.103041

**Published:** 2025-04-09

**Authors:** Raneen Sawaid Kaiyal, Hala Aslih, Inshirah Sgayer, Lior Lowenstein, Ekaterina Shlush

**Affiliations:** a Department of Obstetrics and Gynecology, Galilee Medical Center, Nahariya, Israel; b Department of Obstetrics and Gynecology, Nazareth Hospital EMMS, Nazareth, Israel; c Azrieli Faculty of Medicine, Bar Ilan University, Safed, Israel

**Keywords:** Male infertility, Azoospermia, Chronic kidney disease, End-stage kidney failure, Transplant, Hypogonadism

## Abstract

Azoospermia, the absence of sperm in the ejaculate, affects 1 % of men, with non-obstructive azoospermia (NOA) comprising 60 % of cases. We report a 29-year-old male with NOA and primary infertility, initially asymptomatic with history of chronic diseases and normal evaluations. Routine blood tests revealed elevated creatinine, leading to a diagnosis of end-stage renal disease (ESRD) due to focal segmental glomerulosclerosis. After hemodialysis and kidney transplantation, semen analysis showed cryptozoospermia, suggesting partial spermatogenesis recovery. This case highlights NOA as a potential silent indicator of systemic disease, emphasizing the importance of thorough evaluation and a multidisciplinary approach in idiopathic cases.

## Highlights

• Infertility is a significant healthcare concern beyond reproduction.
• Managing male infertility should address overall health and life expectancy.
• Comorbidities linked to infertility impact both reproductive and general health.
• Recognizing these comorbidities-infertility interactions will enhance patient care and therapeutic advancements.

## Abbreviations

NOA: non-obstructive azoospermia; ESRD: end-stage renal disease; IVF: in vitro fertilization; ICSI: intracytoplasmic sperm injection.

## 1 Introduction

Azoospermia, defined as the complete absence of sperm in the ejaculate, impacts about 1 % of the overall male population and is found in 10–15 % of men facing infertility issues.^1^ Azoospermia is primarily classified to two types: obstructive and non-obstructive.^2^ Obstructive azoospermia, which accounts for about 40 % of the prevalence, occurs when normal spermatogenesis is impaired due to congenital or acquired obstruction of the male reproductive tract.^3^ Non-obstructive azoospermia (NOA), which results from intrinsic spermatogenic failure, accounts for about 60 % of the prevalence.^4^

NOA is associated with multiple etiologies, including hormonal imbalances, genetic abnormalities, toxin exposure, varicocele, infections, inflammation, trauma, and, most frequently, idiopathic causes.^5^ NOA can be linked to various systemic diseases, such as thyroid disorders, diabetes mellitus, chronic liver diseases, and chronic kidney disease, including end-stage renal disease (ESRD).^6^

Chronic kidney disease, particularly ESRD, represents a significant systemic condition with profound effects on male reproductive health, including NOA. The interplay between ESRD and NOA remains poorly understood. The underlying mechanisms are likely multifactorial, involving hormonal dysregulation, oxidative stress, and vascular injury. Moreover, the systemic inflammation, uraemia, and metabolic disturbances associated with ESRD further exacerbate testicular dysfunction and spermatogenic failure. The resulting impact on fertility is significant, with a high prevalence of NOA reported among men with ESRD. Additionally, renal replacement therapies, such as hemodialysis and kidney transplantation, introduce further complexity. While these therapies may partially restore hormonal balance and fertility potential, they pose risks of gonadotoxicity and immunosuppressive-related complications.^7^

Despite the above, ESRD rarely presents with infertility as its sole clinical manifestation. Here, we present a rare presentation of ESRD in a young man with infertility, whose only presenting symptom of ESRD was NOA. Due to the uniqueness of this presentation we aimed to explore the relation between azoospermia and ESRD, with a focus on identifying the pathophysiological mechanisms underlying this association. By integrating clinical findings with molecular insights, we sought to provide a comprehensive understanding of how ESRD contributes to spermatogenic failure and infertility in men. This knowledge is crucial for improving fertility outcomes and guiding management strategies for men with azoospermia and ESRD.

## 2 Case presentation

A young couple with primary infertility presented to our fertility clinic seeking treatment after one year of unsuccessful attempts to conceive. Their medical history was unremarkable for any specific pathology.

The female partner, a 25-year-old, was healthy with no history of chronic diseases, surgeries or ongoing medical treatments. She had never been pregnant and reported regular menstrual cycles without dyspareunia, dysmenorrhea or any symptoms suggestive of endometriosis. She had no history of pelvic infections or sexually transmitted diseases, and no family history of infertility or genetic disorders, including conditions such as autism or intellectual disability. The male partner, a 29-year-old schoolteacher, was healthy and with no history of medical illnesses, surgeries or allergies. He reported smoking but not the use of drugs or alcohol, or any over-the-counter medication or supplements. His family history was unremarkable for infertility, intellectual disabilities or any specific pathology.

The physical examination of the female partner revealed no abnormalities. Her body mass index was within the normal range, and pelvic and reproductive tract ultrasounds were unremarkable. Similarly, the male partner presented with a normal body mass index, typical male hair growth, and an unremarkable physical examination, including a normal urogenital assessment.

Both partners underwent laboratory testing, which demonstrated normal hormonal profiles. Semen analysis of the male partner revealed azoospermia despite normal seminal parameters, including volume, viscosity, pH, colour, leukocyte count and round cell count. He was advised to cease smoking and to repeat the semen analysis after one month. The second analysis yielded identical results, further suggesting non-obstructive azoospermia.

The male partner was referred to a urologist and genetic counselling for further evaluation. The urological examination was unremarkable. The genetic analysis showed a normal 46XY karyotype without Y-chromosome microdeletions. Subsequently, the family physician ordered routine blood tests in preparation for potential surgical sperm retrieval. The blood tests revealed elevated creatinine levels (4.2 mg/dL) and anemia (hemoglobin 9.9 g/dL). This was a significant finding, as previous blood tests performed one decade earlier had shown normal creatinine levels.

Following the above findings, the male partner was referred to a nephrologist and diagnosed with ESRD and nephrotic syndrome, which were attributed to focal segmental glomerulosclerosis. He was initiated on hemodialysis and placed on the national kidney transplant waiting list. Six months later, he underwent a successful kidney transplant, with the donor being his brother. Post-transplant, the patient was started on immunosuppressive therapy to prevent graft rejection.

Three months post-transplant, a semen analysis revealed the presence of a few sperm cells in the ejaculate, consistent with cryptozoospermia. Six months post-transplant, the patient underwent a second semen analysis, which showed results consistent with the three-month post-transplant findings—cryptozoospermia. In accordance with the couple's wishes, no fertility treatment has been initiated thus far, as they are currently hoping for a spontaneous pregnancy. They are, however, well-informed of the potential need for assisted reproductive technologies such as in vitro fertilization (IVF) and intracytoplasmic sperm injection (ICSI) should natural conception not occur.0.

## 3 Discussion

Azoospermia, specifically NOA, is a well-documented consequence of ESRD. This case report highlights the unique presentation of NOA as the sole symptom leading to the diagnosis of ESRD caused by focal segmental glomerulosclerosis. While NOA is associated with systemic conditions such as chronic kidney disease, diabetes and hypertension, its presentation in the absence of overt renal symptoms is rare and diagnostically challenging.

**Figure_1:**
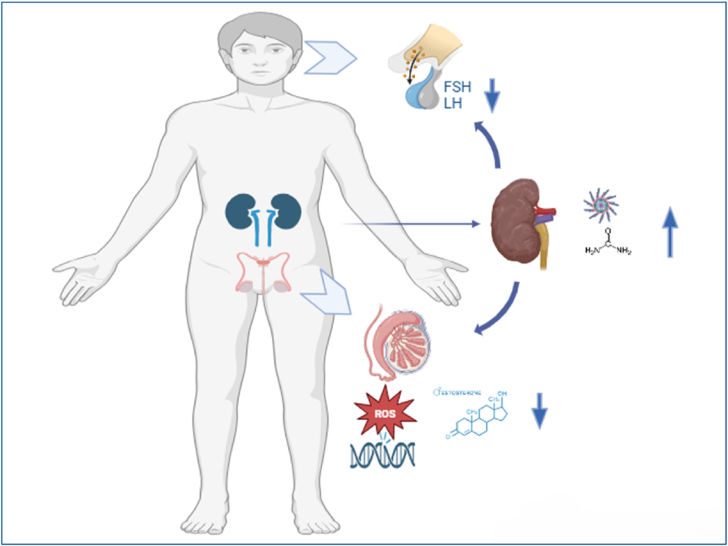
**Non-obstructive Azoospermia** presented as primary infertility. **Systemic Investigation** leading to the diagnosis of **End-Stage Renal Disease** due to **Focal Segmental Glomerulosclerosis. Mechanisms linking ESRD and NOA**: **(1)Hormonal dysregulation** (disruption of the HPG axis, testosterone deficiency). **(2)Oxidative stress & inflammation** (impacting spermatogenesis). **Uraemia & vascular injury** (testicular dysfunction). **(3) Clinical Implications: Non-obstructive Azoospermia** is a potential early marker of systemic disease. Approach for optimal diagnosis and management.

Azoospermia in ESRD is multifactorial, involving hormonal dysregulation, oxidative stress, systemic inflammation and uraemia (Fig. 1). A disrupted hypothalamic-pituitary-gonadal axis is known as a contributing factor, characterized by abnormal release of gonadotropin-releasing hormone and elevated luteinizing hormone.^8^ The exact mechanism is unknown. This disruption, together with chronic inflammation, causes reduced testosterone levels. Leydig cell dysfunction further exacerbates testosterone deficiency.^9^ ESRD is also associated with elevated prolactin due to impaired feedback mechanisms and decreased renal clearance, and also diminished anti-Müllerian hormone, which indicates Sertoli cell defects together with Leydig cell suppression.^10^

Uraemia significantly impairs male fertility through multifactorial mechanisms, including disrupted spermatogenesis, erectile dysfunction and hormonal imbalances.^11^ Men with advanced ESRD often exhibit reduced semen volume, oligoasthenozoospermia and testicular atrophy. Long-term dialysis further decreases testicular volume and germ cell proliferation. Reductions by about 50 % have been reported in sperm viability, motility and morphology. The decline in motility has been shown to correlate with the duration of dialysis.^12^

Kidney transplantation is recognized as a potential intervention to partially restore fertility in persons with ESRD. Post-transplant normalization of testosterone and reductions in levels of luteinizing hormone and follicle-stimulating hormone have been reported.^13^ Improvements in semen parameters, including sperm concentration, motility and morphology, have also been documented, although not universally observed.^14^ In our patient, a subsequent semen analysis at six months post-transplant confirmed the persistence of cryptozoospermia, supporting the notion of sustained, albeit limited, spermatogenic activity following renal transplantation. This aligns with studies that demonstrated that while renal transplantation can reverse some uraemic effects on fertility, persistent testicular fibrosis and maturational arrest may limit complete recovery.

The role of immunosuppressive therapy in transplant recipients is a critical consideration. Calcineurin inhibitors, sirolimus and other agents used to prevent graft rejection can exert gonadotoxic effects and potentially affect sperm quality. Nevertheless, improvements in fertility outcomes after transplantation suggest that these effects may be less significant than those of untreated ESRD^15^

## 4 Conclusion

This report highlights the critical intersection between male infertility and systemic disease, emphasizing the importance of considering renal dysfunction in the differential diagnosis of non-obstructive azoospermia. The unique presentation of azoospermia as the sole manifestation of ESRD underscores the need for comprehensive evaluations in men with azoospermia, even in the absence of other symptoms. Kidney transplantation offers a potential pathway for partial restoration of spermatogenesis and fertility, as evidenced by the detection of cryptozoospermia in the described patient. However, persistent challenges remain due to the multifactorial impact of ESRD, dialysis and immunosuppressive therapy on male reproductive health.This report serves as a reminder for clinicians to adopt a multidisciplinary approach to infertility—one that integrates systemic evaluation with reproductive management—to optimize both general health and fertility outcomes. Although ESRD is rare among men with NOA, this case highlights the importance of including renal function testing, such as a basic metabolic panel, in the initial diagnostic work-up of idiopathic NOA to screen for potential underlying renal disease. Furthermore, our report highlights the need for ongoing research into the mechanisms linking renal failure and infertility, and for the optimization of fertility-preserving strategies in transplant recipients.

## Attestation statement

Data will be made available to the editors of the journal for review or query upon request pre and/or post publication.

## CRediT authorship contribution statement

**Raneen Sawaid Kaiyal:** Writing – original draft, Conceptualization. **Hala Aslih:** Data curation. **Inshirah Sgayer:** Data curation. **Lior Lowenstein:** Supervision. **Ekaterina Shlush:** Supervision.

## Disclosure statement

All authors report no conflict of interest.

## Funding statement

This research did not receive any specific grant from funding agencies in the public, commercial, or not-for-profit sectors.
